# Gram-Negative Bacterial Sensors for Eukaryotic Signal Molecules

**DOI:** 10.3390/s90906967

**Published:** 2009-09-02

**Authors:** Olivier Lesouhaitier, Wilfried Veron, Annelise Chapalain, Amar Madi, Anne-Sophie Blier, Audrey Dagorn, Nathalie Connil, Sylvie Chevalier, Nicole Orange, Marc Feuilloley

**Affiliations:** Laboratory of Cold Microbiology – Signals and Micro-Environment, UPRES EA 4312, University of Rouen, 55 rue Saint Germain, 27000 Evreux, France; E-Mails: wilfried.veron@etu.univ-rouen.fr (W.V.); annelise.chapalain@etu.univ-rouen.fr (A.L.); amar.madi@etu.univ-rouen.fr (A.M.); anne-sophie@etu.univ-rouen.fr (A.S.B.); audrey.dagorn@etu.univ-rouen.fr (A.D.); nathalie.connil@univ-rouen.fr (N.C.); sylvie.chevalier@univ-rouen.fr (S.C.); nicole.orange@univ-rouen.fr (N.O.); marc.feuilloley@univ-rouen.fr (M.F.)

**Keywords:** pathogens, sensors, *pseudomonas*, neurotransmitters, cytokines, hormones, host-pathogen interactions, virulence

## Abstract

Ample evidence exists showing that eukaryotic signal molecules synthesized and released by the host can activate the virulence of opportunistic pathogens. The sensitivity of prokaryotes to host signal molecules requires the presence of bacterial sensors. These prokaryotic sensors, or receptors, have a double function: stereospecific recognition in a complex environment and transduction of the message in order to initiate bacterial physiological modifications. As messengers are generally unable to freely cross the bacterial membrane, they require either the presence of sensors anchored in the membrane or transporters allowing direct recognition inside the bacterial cytoplasm. Since the discovery of quorum sensing, it was established that the production of virulence factors by bacteria is tightly growth-phase regulated. It is now obvious that expression of bacterial virulence is also controlled by detection of the eukaryotic messengers released in the micro-environment as endocrine or neuro-endocrine modulators. In the presence of host physiological stress many eukaryotic factors are released and detected by Gram-negative bacteria which in return rapidly adapt their physiology. For instance, *Pseudomonas aeruginosa* can bind elements of the host immune system such as interferon-γ and dynorphin and then through quorum sensing circuitry enhance its virulence. *Escherichia coli* sensitivity to the neurohormones of the catecholamines family appears relayed by a recently identified bacterial adrenergic receptor. In the present review, we will describe the mechanisms by which various eukaryotic signal molecules produced by host may activate Gram-negative bacteria virulence. Particular attention will be paid to *Pseudomonas*, a genus whose representative species, *P. aeruginosa*, is a common opportunistic pathogen. The discussion will be particularly focused on the pivotal role played by these new types of pathogen sensors from the sensing to the transduction mechanism involved in virulence factors regulation. Finally, we will discuss the consequence of the impact of host signal molecules on commensally or opportunistic pathogens associated with different human tissue.

## 1. Introduction

The number of microorganisms that live inside or on the body of a healthy human adult is estimated at least 10 times greater than the number of human somatic and germ cells [1]. By the end of 2007, the study of this important community was at the origin of the Human Microbiome Project, with the goal of identifying and characterizing all microorganisms found in association with both healthy and diseased humans [2]. The composition of this bacterial community is the result of the combined effects of a selection pressure due to the competition between microbial species, to bacterial strategies of adaptation to the host but also of the host response itself [3–5]. It is then obvious that communication is the key factor regulating this complex ecological system. Besides quorum sensing factors exchanged between bacterial strains or species, bacteria produce virulence factors against the host. Meanwhile, defensins or cytokines are produced by the host in response to the microbial attack, even in the cases where bacteria and eukaryotic cells evolve in perfect harmony. Consequently the two cell types are continuously exposed to signals that can be sensed by both partners.

The ability of a bacterium to persist on or in its host requires that it develops a strategy of tolerance to remain non detectable. Bacteria can modify the host membrane to remain adherent but not engulfed in lysosome [6], or modify the cytoskeleton of the host cell in a way that promotes their own uptake [7], or even exploit innate immune receptors to penetrate into the host cell where they are protected from destruction [8]. Host defense against bacteria depends on innate or acquired immune recognition of the micro-organisms through pattern recognition receptors targeted towards surface (lipopolysaccharides – LPS, peptidoglycans, flagellin, porins, etc.) or inner (DNA, enzymes, toxins, etc.) determinants [9]. In most cells, identification of the bacterial molecules is achieved through membrane toll-like receptors (TLRs) and cytosolic nucleotide oligomerization domain proteins (NOD) [9,10]. Subtype 4 Toll-like receptors (TLR4) that bind to LPS, the endotoxin of Gram-negative bacteria, represent the principal sensing pathway involved in the detection of these micro-organisms. Stimulation of TLR4 initiates the production of pro-inflammatory cytokines and chemokines that recruit and activate leukocytes [11,12] which then activates the whole inflammatory cascade in response to bacterial pathogens [13]. Interestingly, LPS is not an invariant structure. A unique bacterium expresses multiple forms of LPS that can be rapidly modified in response to environmental pressures [14]. For example, *Salmonella* deacylates and palmitoylates lipid A in response to the host micro-environment, which allow this bacterium to avoid detection through TLR4 [15]. Similarly, *Yersinia* and *Pseudomonas* species also modulate their LPS structures, resulting in diminished TLR4 responses to infection [16,17]. These findings have demonstrated that bacteria can rapidly modulate their surface determinants or the host recognition patterns and then control both constituents of the sensing system.

During the establishment of the infectious process, bacteria are exposed and can detect eukaryotic products. A number of pathogenic bacteria have receptors for plasma proteins [18,19] and for different extracellular matrix proteins [20–22]. Bacterial binding to collagen, fibronectin, and laminin has been proposed as an important mechanism for bacterial adherence to target tissues [22,23]. This binding is often, if not systematically, associated to metabolic changes of the micro-organism. The principal mechanism by which bacteria detect and respond to variations of environmental conditions is the two-component signal transduction system [24,25] but other sensory mechanisms are also expressed by bacteria [26–29]. Indeed, bioinformatic studies have made it possible to identify in the genome of prokaryotes sequences showing homology with all great families of eukaryotic receptors (*i.e.* kinase receptor, seven transmembrane receptor, ion channel receptors, etc.) [26,28–32], suggesting that bacteria could express sensors for a multitude of eukaryotic signal molecules including cytokines, neurotransmitters and hormones.

Among these eukaryotic signal molecules, it was recently demonstrated that bacteria express adrenergic-like receptors/sensors able to recognize specifically host epinephrine and nor-epinephrine [33]. This discovery is the accomplishment of works started in the beginning of the 1990s, showing that bacteria are sensitive to stress-related neuroendocrine hormones produced by the host [34,35]. The study of the mechanisms by which stress influences the pathogenesis of infectious disease has allowed the emergence of a new transdisciplinary discipline named microbial endocrinology [36–38].

Since Lyte and Ernst’s precursor work, the bacterial sensitivity to eukaryotic signal molecules appears much wider than initially suspected. In addition to the stress neurohormones [34,39], immunomodulatory peptides are sensed by bacteria [40,41]. Infectious agents are also influenced by other classical eukaryotic hormones as insulin [42], somatostatin [43] or natriuretic peptides [44]. However, pathogens not only express sensors for host molecules but also use these sensors to increase the probability of success of their infectious program.

The neurohumoral environment of the host being an unexpected regulatory network controlling bacterial virulence, microbial endocrinology is now an essential domain of investigation to understand the precise mechanisms at the centre of the pathogenic process. In this review we attempted to analyse the more recent data concerning the physiological effects of eukaryotic signal molecules on Gram-negative bacteria, the nature of these bacterial sensors involved in recognition and their implication in the regulation of virulence.

## 2. Discussion

### 2.1. Gram-negative Bacteria and Neurotransmitters

Stress-related neuroendocrine hormones and more specifically catecholamines have been suspected for a long time to interact with prokaryotes. Indeed, as early as 1930, almost immediately following its first use as therapeutics, cases of adrenaline-associated sepsis were reported [45]. From this time to 1992, the most widely accepted theory to explain the ability of hormones to influence the course of infection involves the suppression of the immune system [36]. The hypothesis that the infectious microorganism itself might be responsive to the host's neuroendocrine environment appeared in 1992, by using a serum-based medium to demonstrate that exposure to catecholamines induced log-fold increases in growth of a limited number of Gram-negative pathogens [34]. This date marked the emergence of an interdisciplinary field, in frontiers of both neuroendocrinology and microbiology and then named microbial endocrinology [36,46].

The examination of a much larger set of clinical isolates by Freestone *et al.* showed that recognition of catecholamines was widespread amongst Gram-positive and Gram-negative bacteria [47]. The ability of norepinephrine, epinephrine and dopamine to enhance the growth of *Escherichia coli*, *Yersinia enterocolitica* and *Pseudomonas aeruginosa* was observed to be dependent on the bacterium species or strain showing marked preference for one or more of the catecholamines [34]. However, norepinephrine was consistently observed to possess the greatest increase in bacterial growth for all strains tested.

Subsequent reports have extended the range of stress hormone-responsive bacteria [48], as well as demonstrated further roles for catecholamines in the production of virulence-associated factors such as toxins [49] and adhesins [50], biofilm formation [51], and quorum sensing [52]. The ability of catecholamines and related inotropes, more precisely norepinephrine, dopamine, and dobutamine (an analogue of dopamine), to increase both bacterial growth and production of virulence associated factors has been demonstrated in a large series of Gram-negative bacteria (Table 1) [47–49,53–55].

The principal mechanism proposed initially to explain the effects of catecholamines was due to the fact that these molecules can bind iron and can be recognized by bacteria as accessory siderophores (iron transporters) [56,57]. However, recently published works [58,59] suggest that the mechanism by which catecholamines act on bacteria is certainly much more complex. The question of a direct receptor-mediated recognition of catecholamine by bacteria as a communication signal is still controversial. It was first reported that neither α- nor β-adrenergic receptor agonists or antagonists can affect bacterial growth as observed with norepinephrine [35], suggesting the absence of adrenergic receptor-like mediated system. However a recent report using a wide spectrum of adrenergic and dopaminergic antagonists confirmed the presence of specific recognition systems for norepinephrine, epinephrine and dopamine [58].

During study of the activator of the quorum-sensing *E. coli* regulator A (QseA), QseC/QseB, a two-component regulatory system, was identified [60,61]. Interestingly, the activators of this regulatory system, which represent a type of sensor, can be from prokaryotic origin (quorum sensing signal autoinducer AI3) but also from eukaryotic origin (epinephrine/norepinephrine) [33,52,62]. Binding and pharmacological studies suggest now that the QseC sensor kinase represents a bacterial analog of a eukaryotic adrenergic receptor [33,52,63].

The QseC/QseB system belongs to the family of the bacterial two-component systems. QseC senses the epinephrine or the norepinephrine and phosphorylates its associated transcription factor QseB, which finally regulates expression of bacterial virulence genes [33,64]. More detailed molecular analysis of the QseC signaling mechanism has shown that this sensor activates the expression of another two component signal system named QseE/QseF [64]. This new system should represent a second bacterial adrenergic receptor subfamily specifically activated by epinephrine but not by AI-3 [65]. It appears that, as in mammals, different sensors for a same eukaryotic molecule can co-exist in a bacterium and should be coupled to different bacterial physiological responses. Bioinformatic screening of *E. coli* QseE analogs have made it possible to identify the presence of proteins related to this sensor in 25 important human and plant bacterial pathogens [64], including *Shigella flexneri* [64], *Salmonella enterica* Typhimurium [66,67] and *P. aeruginosa* [64]. *In vivo* studies showing that *qseC* mutants have lower virulence in rabbits (*E. coli* mutants) and in pigs (*S. enterica* mutants) [33,66], confirm the probable involvement of bacterial adrenergic sensor(s) in the global virulence of many pathogens.

In *P. aeruginosa* norepinephrine enhances growth through a reduction of the lag time [47,55]. Norepinephrine and dopamine are more effective than epinephrine and isoproterenol (β1- and β2-adrenoreceptor agonists) [68] but the detection pathway was not studied in detail. It was only shown that, in *P. aeruginosa,* norepinephrine increases the expression of the PA-I lectin by the bacterium [69]. This complex plays a key role in the pathogenicity of *P. aeruginosa* in the intestinal epithelium [70]. In addition, *P. aeruginosa* express a two-component regulator system signal sensor kinase called PmrB that shows significant homology with the *E. coli* catecholamine sensor QseC [64]. As *P. aeruginosa* is a minor intestinal commensal [71] and that surgical stress increases intestinal norepinephrine release [69] changes in the micro-environment of the bacterium could result in unexpected variations of virulence. Gamma aminobutyric acid (GABA) represents the most important inhibitory neurotransmitter in animals [72]. In micro-organisms, GABA has been during a long time, considered only as a nutriment [73,74]. However, the presence in bacteria of enzymes implicated in the GABA metabolism allows the synthesis of this neurotransmitter by prokaryotes [75,76]. It was even shown that bacteria are able to release GABA [77,78], suggesting that, as in eukaryotes, GABA should represent a communication molecule. The physiological relevance of GABA-release by bacteria was demonstrated by showing that GABA from diverse micro-organisms rapidly and synchronously induce planktonic larvae of the red abalone, *Haliotis rufescens*, to settle and commence behavioral and developmental metamorphosis [78–80].

The molecular mechanism involved in bacterial uptake of GABA has been characterized and a possible direct action of GABA on micro-organisms has been evaluated. The presence of a bacterial GABA binding protein (GBP) was first demonstrated in *Pseudomonas fluorescens* [81]. The GBP was partially purified and was identified as a 42 kDa periplasmic protein [82]. This bacterial GBP shows high affinity and specificity for GABA, additionally it is saturable, sodium-independent and competitively inhibited by muscimol (a potent GABA analogue) [81]. This GBP therefore shares common characteristics with the mammalian GABA_A_-receptor for GABA [83] strongly suggesting that GABA should be considered as a bacterial communication molecule. The bacterial GBP appears to be present in other micro-organisms, such as the plant pathogen *Agrobacterium tumefaciens*, in which this GABA sensor was even recently cloned, purified and crystallized [84]. In *A. tumefaciens*, GABA produced by plants after injury, modifies the production of the bacterial quorum sensing molecule, which thereby modulates *A. tumefaciens* virulence [85]. This result provides evidence for the inter-kingdom communication function of GABA between eukaryotes and bacteria. The study of the direct impact of GABA on bacteria has shown that this neurotransmitter influences transcriptional activities in *Pseudomonas aeruginosa* [86], suggesting that animal or human pathogens such as *P. aeruginosa* are also sensitive to GABA. This hypothesis was confirmed by the observation that exogenous GABA modulates the global virulence profile of both *P. aeruginosa* and *P. fluorescens* toward animal cells *in vitro* [87].

### 2.2. Gram-negative Bacteria and Immune System Communication Molecules

Cytokines are polypeptide signal molecules mediating a multitude of physiological responses to infection and particularly inflammation. Their actions are relayed by a cascade of biological events leading to propagation of inflammatory response and host defense against pathogens. The natural role of these molecules, when they are present at the right concentration, is positive for the host since they recruit and activate immune cells so as to eradicate the germs at the origin of the infectious process. In addition to this well documented role of cytokines, another parallel action during the infectious process was suspected at the beginning of the 1990s. Indeed, it was shown that stress can promote massive translocation of intestinal bacteria and/or toxins into the systemic compartment [88]. This mechanism provokes the release of pro-inflammatory cytokines and other signaling molecules which participate in the systemic inflammatory response syndrome. Bacteria then come in to direct contact with these eukaryotic molecules. These observations were at the origin of the study of the effect of cytokines on bacteria.

The direct impact of cytokines on bacteria was strongly investigated *in vitro* (Table 2) and was led to the observation that, interleukin-1 [40], interleukin-2 [89], granulocyte-macrophage colony-stimulating factor [89], interleukin-6 [90], interferon-γ [41] and tumor necrosis factor α [91] all promote bacterial growth. Only, IL-4 was without effect on bacterial growth [89]. It does appear that cytokines have a dual effect on inflammation and infection; pro-inflammatory cytokines act as stimulators of bacterial growth when they reach a sufficient concentration [92].

The effect of cytokines involves the presence of membrane structures at the surface of bacteria to allow detection of these polypeptide eukaryotic molecules. This hypothesis was essentially investigated by binding experiments using radiolabeled ligands. It was shown that interleukin-1 β (IL-1 β) binds to virulent *Escherichia coli* strains in a saturable way [40]. The fact that IL-1ra, a specific IL-1 receptor antagonist, inhibited the binding of IL-1 β was consistent with the presence of a functional IL-1-like receptor structure on this bacterial strain [40]. The search for the bacterial sensor of IL-1 β led to the identification of Caf1A (capsule antigen F1 assembly) a protein from *Yersinia pestis* which is the target of IL-1 β in this species [93]. The high specificity and affinity of IL-1 β to Caf1A (Kd = 1.40 × 10^−10^ M) enabled this protein to be designated as a real IL-1 β bacterial receptor [93]. The localization of Caf1A in the outer membrane of the bacterium is in harmony with a role of sensor. Caf1 A is then characterized by a double function, the first identified in capsule antigen F1 assembly and the second in the detection of IL-1β. The transduction mechanism which allows for the passage of the signal detected in the outer bacterial membrane to induction of bacterial growth remains to be investigated. Additionally Caf1 A is the unique bacterial sensor for human IL-1β identified until now. Furthermore, while it was also suggested that the IL-1 β bacterial sensor should be involved in the regulation of bacterial pathogenicity [40] this activity was never directly demonstrated.

Another cytokine, TNF-α, can also bind to a great diversity of Gram-negative bacteria [91]. The sensor for TNF-α was investigated in detail in the bacterial pathogen *Shigella flexneri*. This receptor is expressed at an average of 276 copies per bacterium and its Kd measured using ^125^I-labeled TNF-α is about 2.5 nM [91]. The pharmacological properties of the bacterial TNF-α receptor are clearly different from its eukaryotic counterpart [94]. For instance, the binding of labeled TNF-α to the bacterial receptor can be inhibited by cold TNF-α but not by cold TNF-β. Moreover monoclonal antibodies to the 55-kDa or 75-kDa TNF-α receptor, the major forms expressed in eukaryotic cells, have no effect on ^125^I-TNF-α binding to bacteria [91]. As observed with interleukins, TNF-α appears to promote bacterial virulence even though it does not act on bacterial growth. Indeed, pre-treatment of *S. flexneri* with TNF-α increases the invasive potential of the bacteria on HeLa cells [91]. It is interesting to note that this phenomenon is abrogated when HeLa cells are exposed to antibodies against the 55-kDa TNF-α receptor subtype suggesting that TNF-α/TNF-α bacterial receptor complexes should interact with eukaryotic TNF-α receptors. Whereas host cells are known to express receptors recognizing bacteria for the purpose of activating the immune system, this work reveals that bacteria themselves might possess specialized receptors that in turn recognize and respond to host immune activation.

Such bacterial sensors should explain the behavior of opportunistic pathogens that only express virulence in organisms having abnormal immunological or inflammatory status. This is the case of *P. aeruginosa* that can colonize various environmental niches and is only able to infect hosts with defective immune system function [95]. None of the cytokines tested, including human IL-2, IL-4, IL-6, IL-8, IL-10, IL-12, interferon gamma (IFN-γ), and tumor necrosis factor alpha (TNF-α) have any significant effect on growth of *P. aeruginosa* [96]. However, screening of the impact of cytokines on the secretion of virulence factors by this bacterium, revealed that the expression of type I lectin (PA-I lectin), an adhesin essential to the lethality of *P. aeruginosa* [70], and production of the siderophore pyocyanin, are specifically induced by IFN-γ [96]. Concurrently it was demonstrated that the effect of IFN-γ on *P. aeruginosa* is associated with the activation of inter-bacterial Quorum sensing communication [96]. A 35-kD protein was found in the outer membrane of *P. aeruginosa* that binds specifically this cytokine in a dose dependent manner [96]. This protein was identified by electronspray ionization / telomeric repeat amplification liquid chromatography tandem mass spectrometry as the outer membrane porin OprF [96]. This result was confirmed by construction of OprF deletion mutants.

Toxicological studies were at the origin of observations that lead to identification of another family of mediators of inflammation specifically detected by bacteria. There is ample evidence that the prevalence and severity of bacterial infections is increased in injected opiate users [97]. In addition, it is known that following stress, endogenous opioids act as paracrine and autocrine signals with high levels of functional redundancy and pleiotropy [98]. These observations, coupled with the findings that neutrophils themselves can synthesize and release opioids at the inflammation site, strongly suggest that bacteria are exposed to opioids during the course of infection. Experimental studies showed that in mice, stress results in the release of factors, including opioids, into the intestinal lumen which directly activate the virulence of *P. aeruginosa*, leading to disruption in the intestinal epithelial barrier [96]. In order to explain this phenomenon, Alverdy’s group tested the impact of different opioids, with specificity towards µ-, λ- and κ-opioid receptors, on the growth of *P. aeruginosa* PAO1. None of the opioids agonists were active on bacterial growth in PAO1 but it appeared that the synthetic agonist U-50,488 and dynorphin, an endogenous κ-opioid receptor ligand enhanced the virulence of the bacterium [99]. This effect was attributed to an increase of bacterial pyocyanin production [99]. This result is another demonstration that eukaryotic signal molecules can modify the physiology of bacteria without directly affecting their growth rate. The original action of ligands of κ-opioid receptors on *P. aeruginosa* strongly supported the hypothesis that these micro-organisms expressed a specific sensor for opioid-related molecules [99]. The physiological relevance of this hypothesis was studied by an *in vivo* experiment, showing that dynorphin is released into the intestinal lumen after injury and accumulates on desquamated epithelium, where it finally binds to *P. aeruginosa* [99]. The binding of dynorphin to *P. aeruginosa* was confirmed *in vitro* by direct immunostaining using anti-dynorphin antibodies. More precisely, electron microscopic studies revealed that dynorphin was found predominantly in the cytosolic compartment of bacterial cells [99] suggesting that, despite its peptide structure, dynorphin was able to cross the double membrane of *P. aeruginosa*. This unexpected phenomenon requires that dynorphin either binds a membrane sensor which is then translocated into the bacterial stroma or that dynorphin can cross the membranes through porins or transporters before being recognized by a cytoplasmic sensor. The second hypothesis appears more probable, since in eukaryotic cells dynorphin can cross the cytoplasmic membrane through a mechanism which is independent of opioid receptors [100]. Afterwards, dynorphin could act the same way on intra-bacterial opioid receptor-like molecules. Until now the cytoplasmic target of dynorphin in bacteria was not been identified. It was demonstrated that in *P. aeruginosa,* the global virulence regulator Mvfr [101] is involved in the action of dynorphin [99] but it does not appear as the real binding site of dynorphin.

Finally, we can also notice that whereas eukaryotic immune communication molecules can interfere with bacterial virulence by acting on the production of bacterial communication molecules of the quorum sensing, as demonstrated with dynorphin and interferon-γ, the opposite is also true since bacterial communication molecules of the quorum sensing, apart from regulating the expression of virulence factors, can themselves modulate the host immunological system [102–104]. For instance, it appears that in *Pseudomonas*, bacterial signal molecules such as N-(3-oxododecanoyl)-l-homoserine lactone (3oxo-HSL) and Pseudomonas Quinolone Signal (PQS) possess dual activities: inducers of virulence factors but also immune-modulators facilitating the infective properties of the pathogen [105].

### 2.3. Gram-negative Bacteria and Peptide Hormones

The intense use of antibiotics has provoked the emergence of antibiotic-resistant strains of many Gram-negative bacterial species [106], requiring the development of new strategies to treat bacterial infections [107]. In this context, antimicrobial peptides appeared as a promising solution [108, 109]. Antimicrobial host defense peptides are widely produced by animals and plants and represent a very ancient defense mechanism [110]. Most of these peptides have cationic properties that allow interactions with the bacterial cytoplasmic membrane [111–113]. However, bacteria have developed mechanisms to resist antimicrobial peptides [114,115]. These mechanisms have been extensively studied in *Salmonella typhimurium* but are also present in other bacterial pathogens including *Shigella flexneri*, *Erwinia chrysantemi* or *Escherichia coli* [116–118]. For exemple, the PhoP/PhoQ system responds to sublethal concentrations of antimicrobial peptides to promote the resistance of bacteria [113]. It was demonstrated that the enzymatic activity of PhoQ, the sensor kinase of the system, is directly activated by antimicrobial peptides, indicating that sensor kinase present in the cytoplasmic membrane of bacteria may be essential to protecting micro-organisms such as *S. typhimurium* [113] or in *P. aeruginosa* [119,120] from the action of antimicrobial peptides. These observations reveal that bacteria can sense small natural eukaryotic peptides and initiate a transcriptional program that promotes bacterial virulence [113]. To identify natural peptides able to counteract bacterial infection, a series of neuropeptides and peptide hormones were identified which show structural homology with antibacterial peptides (Table 3) suggesting a secondary bactericidal activity for these communication molecules [43,121–126].

A significant percentage of patients who develop melioidosis have preexisting diabetes mellitus, a hypothesis was presented that insulin may modulate the physiology of *Burkholderia pseudomallei*. Further demonstrations led to show that insulin markedly inhibits the growth of *B. pseudomallei* [42]. Binding studies revealed that *B. pseudomallei* express a specific and saturable insulin binding site allowing the simultaneous recognition of 5,000 insulin molecules per bacterial surface [42]. This property of *B. pseudomallei* could explain the influence of serum insulin levels on the pathogenic activity of the bacterium during septicemic infections [42]. It was also suggested that, as in eukaryotes, the insulin receptor expressed by *B. pseudomallei* may be associated to a signal coupling system involving phospholipase and protein tyrosine phosphatase [127]. These results have been questioned since contradictory studies have indicated that the inhibition of insulin on *B. pseudomallei* should be ascribed to the presence of *m*-cresol, a preservative, in the preparation [128]. The explanation of the sensitivity of diabetics to melioidosis remains controversial [129] and further investigations are necessary to confirm the expression of a high-affinity binding site for human insulin on *B. pseudomallei*.

The natural ecological niche of *Helicobacter pylori* is the gastric mucosa, an environment normally hostile to the majority of microorganisms. In the stomach, this human pathogen is in contact with food but also with different hormones involved in gastric regulation such as gastrin and somatostatin. The fact that human gastrin levels are elevated during infection with *H. pylori* and return to basal levels after eradication of the bacterium confirms that *H. pylori* is normally exposed to this human hormone. This interaction should participate in the pathological activity of the bacterium since *in vitro* it has been demonstrated that human gastrin is able to stimulate the growth of *H. pylori* in a specific and dose-dependent manner by shortening the lag time, increasing the growth rate during the logarithmic phase, and rising the final bacterial concentration during the stationary phase [130,131]. These effects were observed over a wide range of concentrations, compatible with the physiological luminal and serum levels of gastrin. The interaction between *H. pylori* and gastrin was confirmed using ^125^I-labeled gastrin and measuring bacterial uptake [131]. Although, it was also shown that cholecystokinin and pentagastrin specifically inhibit the binding of human gastrin on *H. pylori* [131] and only the human sequence of gastrin can stimulate the growth of bacteria [130]. The suspected *H. pylori* gastrin sensor has not yet been identified.

Somatostatin is a polypeptide hormone which also regulates gastric function and cell proliferation [132]. In contrast to the above described effect of gastrin, at the concentration present in the human gastric liquid (10^−11^ mol/L), somatostatin significantly inhibits the proliferation of *H. pylori* [43]. In eukaryotic cells, somatostatin acts through receptors coupled to cyclases activation via membrane associated G proteins [133]. It is interesting to note that, in *H. pylori,* somatostatin increases about 10 times the intra-bacterial concentration of cyclic Guanylate Mono-Phosphate (cGMP) and cyclic Adenosine Mono-Phosphate (cAMP)*,* and that the antiproliferative effect of somatostatin is fully reproduced by use of a stable analogue of cGMP, 8-bromo-cGMP (8BrcGMP) [43]. Scatchard plot showed that *H. pylori* express one class of binding site for somatostatin with relatively high affinity (Kd, 0.31 nmol/L) [43]. A partial pharmacological characterization of this bacterial receptor revealed that the effect of somatostatin is only mimicked by somatostatin receptor sub-type 2 (SSRT-2) analogs and partially blocked by SSRT-2 antibodies [43]. The presence of a bacterial somatostatin sensor showing similarities with the eukaryotic SSRT-2 was clearly suspected. The fact that somatostatin neither binds to *E. coli* nor affects its proliferation, indicates that the expression of this bacterial sensor is restricted to specific bacterial species and suggests that a horizontal transfer occurs between eukaryotic cells and *H. pylori*.

Natriuretic peptides, a family diuretic and osmoregulatory peptides, which possess a structure similar to cationic antimicrobial peptides [134], have been proposed as antimicrobial active peptides to a large spectrum of micro-organisms [135]. More precisely, it was shown that the human Brain Natriuretic Peptide (BNP) is bactericidal at the concentration of 60 μM, whereas other tested peptides were devoid of effect [135]. The fact that C-type natriuretic peptide (CNP) is able to make pores in reconstructed membrane [136] has reinforced the hypothesis of a functional antibacterial activity associated with this peptide family. Nethertheless, both BNP and CNP are present in the plasma at concentrations clearly lower than the ones used to evaluate their potential antibacterial properties (10 pM versus 60 μM) [137]. Furthermore, it was shown that BNP concentration is increased in human blood during septic shock [138,139], and that plasma N-terminal pro-brain natriuretic peptide (NT-proBNP) increases in a model of systemic exposition to *E. coli* endotoxin (or lipopolysaccharide, LPS) in healthy volunteers [140]. It is then certain that pathogen bacteria are exposed to natriuretic peptides in the plasma during infection. In order to determine the real impact of natriuretic peptides on bacterial physiology, in our laboratory we studied the effect of human BNP and CNP, at micromolar range, on a family of pathogens well known for their implication in septic, shock, namely *Pseudomonas.* It was first observed that both human BNP and CNP at micromolar concentration have no effect on *Pseudomonas* growth or cultivability [44,141]. In contrast, exposure of *P. aeruginosa* to BNP and CNP provokes an enhancement of the virulent activity of this pathogen [44]. By homology with eukaryotes, where natriuretic peptides act through receptors coupled to cyclases, it was observed that free permeable stable analogs of cyclic AMP (dibutyry cyclic AMP, dbcAMP) and cyclic GMP (8BrcGMP), mimic the pro-virulent effect of BNP and CNP on *P. aeruginosa*. This observation and the two fold increase of intra-bacterial concentrations of cAMP measured after exposure to CNP strongly suggests the presence of cyclic nucleotide-dependent natriuretic peptide sensor in *Pseudomonas* [44]. It is well known that in *Pseudomonas* the adenylate cyclase CyaB serves as a primary sensor for the assimilation of host-generated signals and that its activation enhances the global virulence of *Pseudomonas* [142–144]. The presence in bacteria of numerous genes coding for membrane adenylate and guanylate cyclases [145] and the involvement of cyclases in the regulation of virulence support the existence of a complex pathway based on cyclic nucleotides and control the infectious potential of bacteria. It is suggested that activation of membrane adenylate and/or guanylate cyclases in *Pseudomonas* could lead to the formation of cAMP or cGMP as second messengers regulating *Pseudomonas* virulence. The intracellular cascade involved in this process remains essentially unidentified. However, it does appear that the protein Vfr, a central cAMP-binding protein, that controls the production of numerous virulence factors in *P. aeruginosa* is also required for the action of natriuretic peptides, suggesting that the sensor for these peptides in *Pseudomonas* is functionally associated to Vfr [44]. The molecular form and the localization of the natriuretic peptide sensors in *Pseudomonas* require further investigations. In eukaryotic cells natriuretic peptide receptors exist under three principal subtypes which are all located in the cytoplasmic membrane. The studies realized in *Pseudomonas* suggest that different forms of natriuretic peptides sensors also exist in bacteria. Indeed, in *P. aeruginosa* natriuretic peptides act preferentially through cAMP [44] whereas in *P. fluorescens* these peptides essentially stimulate the production of cGMP [141]. We can not exclude an action of natriuretic peptides on cytoplasmic cyclases which are also present in prokaryotes [146] since analysis of the structure of CNP and BNP suggest that they could directly cross the cytoplasmic membrane. In contrast with dynorphin [100], such phenomenon have never been reported in eukaryote or prokaryote for natriuretic peptides.

Sensors showing high homology with eukaryotic receptors have been identified in bacteria, even though the exact nature of the ligand still remains uncertain both in eukaryotes and prokaryotes. This is the case of the Translocator Protein (TSPO), previously identified as peripheral-type benzodiazepine receptor, that was initially described in the outer-membrane of mitochondria [147] but was also found in the bacterium *Rhodobacter sphaeroides* [148] and related micro-organisms of the α-subdivision of purple bacteria [149]. As theses organisms likely gave rise to the endosymbiont at the origin of mitochondria, the existence of TSPO in these bacteria was originally considered to be a relic of evolution. However, recent studies indicate that TSPO is widely distributed in prokaryotes, although its presence is irregular even into a same genus [150]. These variations have been ascribed to the presence of an abundance of genes coding transposases at the vicinity of *tspO*, suggesting important but ancient exchanges between bacteria, and may be eukaryotes. In *Rhodobacter* TSPO serves as an oxygen sensor [151], whereas its functions in Gram-negative bacteria such as *P. fluorescens,* is apparently more complex including regulation of adhesion and virulence [150]. Worth mentioning, in *Pseudomonas*, as in mitochondria, TSPO is functionally associated to a membrane porin, although its localization in the membrane and sense of insertion should be notably different [150].

## 3. Conclusions and Perspectives

**Figure 1.**
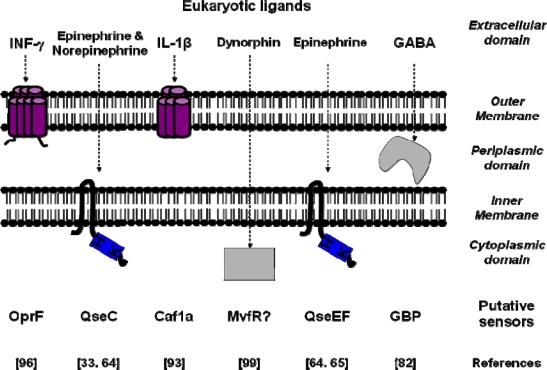
Location of the characterized bacterial sensors for eukaryotic signal molecules. The Gram-negative cell envelope is composed of the periplasmic space confined between the outer membrane and the inner membrane. The cell envelop separates the extracellular domain which contains eukaryotic ligands (top of the figure) and the cytoplasmic domain of the bacteria. The name of the putative sensors and the references associated to their characterization are indicated on the bottom of the figure. GABA: gamma aminobutyric acid.

It appears nowadays that eukaryotic signal molecules have multiple and fundamental effects on bacterial physiology and more particularly on pathogens. The distribution of bacterial sensors appears heterogeneous since they are present in all compartments of Gram-negative bacteria (Figure 1).

However, the whole number of bacterial sensors completely characterized at a molecular level remains limited (Table 4). A reason for this situation should be the difficulty in characterizing bacterial sensors for eukaryotic molecules, especially in the case of neurotransmitters. The identification of a putative receptor-mediated process in bacteria generally starts from clinical or environmental observations where it is difficult to distinguish the role of the prokaryotic and eukaryotic cellular partners. For that reason, the involvement of bacteria in many apparently non-infectious pathologies, including atherosclerosis, Parkinson disease or biliary cirrhosis, remains controversial. The “dogma” of the real sterility of the inner medium in metazoans, including humans, is now even brought forward. A major limitation to the identification of bacteria sensors to eukaryotic messengers is our very limited knowledge of their pharmacology and consequently the quasi absence of chemical tools (specific agonists or antagonists) designed for their study. In parallel, a molecular approach using eukaryotic receptor sequences in order to “fish” for putative bacterial sensors for hormones or neurotransmitters remains speculative in regard to the divergence of these two kingdoms [26,31]. The present review shows the richness and diversity of host-pathogen interactions, and their consequences for human health. Treatments such as, gastric acid suppression, vasoactive agents and opioids among others certainly limit the ability of the intestinal tract to excrete non-commensal pathogens, and artificial nutrition can favor the exposure of pathogens like *P. aeruginosa* to a composite environmental cue that can directly activate its virulence circuitry [96,99,152,153]. In this regard, understanding of the mechanisms by which *Pseudomonas* or other pathogens activate the expression of their virulent profile in response to the stressed host, must be an important tool to anticipate the consequences of these virulence modulations on the normal and pathologic human microbiome. Identification of the sensors involved in these processes is opening a new wide field of promising research. Near the characterization of the binding domains of these bacterial types of sensors, decipher the signal transduction network associated to their activation represent also an important challenge in order to develop therapies to counteract the propagation of pathogen in host.

## Figures and Tables

**Table 1.** Neurotransmitters and bacteria.

**Signal molecules**	**Bacterial species**	**Observed effect**	**References**
Norepinephrine	*Acinetobacter lwoffii*	↑Growth	[47]
	*Bordetella bronchiseptica*	↑Growth	[48]
	*Campylobacter jejuni*	↑Growth, virulence	[154]
	*Citrobacter freundii*	↑Growth	[47]
	*Hafnia alvei*	↑Growth	[47]
	*Morganella morganii*	↑Growth	[47]
	*Proteus mirabilis*	↑Growth	[47]
	*Shigella sonnei*	↑Growth	[55]
	*Vibrio splendidus*	↑Growth	[155]
	*Vibrio parahaemolyticus*	↑Growth, virulence	[156, 157]
	*Xanthomonas maltophilia*	↑Growth	[47]

Catecholamines	*Escherichia coli*	↑Growth	[34, 58]
	*Aeromonas hydrophila*	↑Growth	[54]
	*Pseudomonas aeruginosa*	↑Growth, virulence	[47, 55, 68, 69]
	*Salmonella enterica*	↑Growth, adhesion	[59]
	*Yersinia enterocolitica*	↑Growth	[34, 59]

GABA	*Agrobacterium tumefaciens*	↑Virulence	[85]
	*Pseudomonas aeruginosa*	↑Virulence	[87]

This Table summarizes the different neurotransmitters that were shown to modify directly Gram-negative bacteria physiology, the neurotransmitter-responsive bacteria and the observed effects. GABA: gamma aminobutyric acid.

**Table 2.** Immune modulators and bacteria.

**Signal molecules**	**Bacterial species**	**Observed effect**	**References**
IL-1β	*Escherichia coli*	↑Growth	[40]
	*Acinetobacter* spp	↑Growth	[90]

TNF-α	*Shigella flexneri*	↑Invasion	[91]
IL-2	*Escherichia coli*	↑Growth	[89]
GM-CSF	*Escherichia coli*	↑Growth	[89]
IL-6	*Pseudomonas aeruginosa*	↑Growth	[90]

INF-γ	*Klebsiella pneumoniae*	↑Growth	[41]
	*Escherichia coli*	↑Growth	[41]
	*Pseudomonas aeruginosa*	↑QS/virulence	[96]

Dynorphin	*Pseudomonas aeruginosa*	↑QS/virulence	[99]

This Table summarizes the different immune modulators that were shown to modify directly Gram-negative bacteria physiology, the immune modulator-responsive bacteria and the observed effects. QS: Quorum sensing, GM-CSF: Granulocyte Macrophage Colony Stimulating Factor.

**Table 3.** Peptide hormones and bacteria.

**Signal molecules**	**Bacterial species**	**Observed effect**	**References**
Substance P	*Escherichia coli*	Antibacterial	[125]
	*Pseudomonas aeruginosa*	Antibacterial	[158]

Insulin	*Burkholderia pseudomalei*	↓Growth	[42]
CGRP	*Escherichia coli*	Antibacterial	[126]
Chromogranins	*Escherichia coli*	Antibacterial	[122]
Somatostatin	*Helicobacter pylori*	↓Growth	[43]

hBNP	*Pseudomonas aeruginosa*	↑Virulence	[44]
	*Pseudomonas fluorescens*	↑Virulence	[141]
	*E. coli; P. aeruginosa*	Antibacterial	[135]

CNP	*Pseudomonas fluorescens*	↑Virulence	[141]
	*Pseudomonas aeruginosa*	↑Virulence	[44]

Gastrin	*Helicobacter pylori*	↑Growth	[131]
ACTH	*Vibrio splendidus*	↑Growth	[155]
Neuropeptide Y	*Escherichia coli*	Antibacterial	[125]

This Table summarizes the different peptide hormones that were shown to modify directly Gram-negative bacteria physiology, the peptide hormone-responsive bacteria and the observed effects. hBNP: human brain natriuretic peptide, CNP: c-type natriuretic peptide, CGRP: Calcitonin gene related peptide, ACTH: Adrenocorticotropic.

**Table 4.** Bacterial sensors for eukaryotic signal molecules.

**Signal molecules**	**Putative sensors**	**Binding Site affinity**	**Bacterial species**	**References**
IL-1β	Caf1A	Kd=0.14 nM	*Yersinia pestis*	[93]
TNF-α	-	Kd=2.5 nM	*Shigella flexneri*	[91]
Epinephrine/Norepinephrine	QseC		*Escherichia coli*	[33]
Epinephrine	QseE/QseF		*Escherichia coli*	[64,65]
Somatostatin	SSRT2-like	Kd=0.3 nM	*Helicobacter pylori*	[43]
INF-γ	OprF		*Pseudomonas aeruginosa*	[96]
Dynorphin	Mvfr		*Pseudomonas aeruginosa*	[99]
GABA	GBP	Kd=65 nM	*Pseudomonas fluorescens*	[82]

This Table lists the putative Gram-negative bacteria sensors for eukaryotic signal molecules identified until now, their binding affinity and the species where they were found. GABA: gamma aminobutyric acid.
